# Inter-institutional variations in oxytocin augmentation during labour in German university hospitals: a national survey

**DOI:** 10.1186/s12884-019-2348-x

**Published:** 2019-07-09

**Authors:** Sonja Helbig, Antje Petersen, Erika Sitter, Deirdre Daly, Mechthild M. Gross

**Affiliations:** 1 Midwifery Research and Education Unit, Department of Obstetrics, Gynaecology & Reproductive Medicine, Hannover Medical School, Carl-Neuberg-Str. 1, D – 30625 Hannover, Germany; 2 School of Nursing and Midwifery, Trinity College Dublin, 24 D’Olier Street, Dublin, D02 T283 Ireland

**Keywords:** Oxytocin, Guideline, Germany, Labour, Augmentation, Midwifery

## Abstract

**Background:**

There are several international guidelines on oxytocin regimens for induction and augmentation of labour, but no agreement on a standardised regimen in Germany. This study collated and reviewed the oxytocin regimens used for labour augmentation in university hospitals, with the long-term aim of contributing to the development of a national clinical guideline.

**Methods:**

Germany has 34 university hospital compounds, representing 39 maternity units. In this observational study we asked units to provide standard operational procedures on oxytocin augmentation during labour or provide the details in a structured survey. Data were collected on the dosage of oxytocin, type and volume of solutions used, indications and contraindications for use and discontinuation, case-specific administration, and on who developed the procedures. Findings were analysed descriptively.

**Results:**

A total of 35 (90%) units participated in this study. Standard operating procedures were available in 24 units (69%), seven units (20%) did not have procedures and information was missing from four units (11%). Midwives participated in the development of standard operating procedures in 15 units (43%). Infusions were most commonly prepared using six units of oxytocin in 500 ml 0.9% normal saline solution (12 mU/ml). The infusions were started at 120 mU/hour and increased by 120 mU/hour at 20-min intervals up to a maximum dosage of 1200 mU/hour. The most common indication for use was delayed progress in labour. Infusions were stopped when uterine contractions became hypertonic and/or the fetal heart rate showed signs of distress. Most of the practices described aligned with international guidance. All units used reduced oxytocin dosages for women with a history of previous caesareans section, as recommended in the international guidelines, and restrictive use was advised in multiparous women. The main difference between units related to combined use of amniotomy and oxytocin, recommended by three guidelines but used in only four maternity units (11%).

**Conclusions:**

While there was considerable variation in the oxytocin augmentation procedures, most but not all practices used in these 35 German maternity units were comparable. Establishing a national guideline on the criteria for and administration of oxytocin for augmentation of labour would eliminate the observed differences and minimise risk of administration and medication error.

**Electronic supplementary material:**

The online version of this article (10.1186/s12884-019-2348-x) contains supplementary material, which is available to authorized users.

## Introduction

There are several international guidelines on oxytocin regimens used to induce or augment labour, but no agreement on a standardised regimen in Germany. Different guidelines and publications report the use of low-dose and high-dose regimens, and variations in regimens used the same maternity unit depending on a practitioner’s preferences [1–3]. Such variations, at national and institutional level can be confusing and increase the risk of errors and harms occuring [3, 4]. This study collated and compared the standard operating procedures (SOPs) used for augmentation of labour in 35 of the 39 university-based maternity units in Germany.

## Background

In 1906, Sir Henry Dale showed that the hormone oxytocin, in the form of a dried ox-pituitary extract, promoted uterine contractions [5]. Oxytocin is a peptide with nine amino acids, only two of which differ from the hormone arginine vasopressin (AVP) [6], and was first synthesised in 1953 by Vincent du Vigneaud et al. [7]. Due to the high structural similarity of oxytocin and AVP, both hormones signal through G-protein-coupled receptors, certain cross-reactivity properties are evident [8]. Both hormones cause muscle contractions, and are associated with sexuality, anxiety, depression, cancer and social behaviour [9–11]. While AVP is known to, mainly, regulate vasoconstriction [12], oxytocin is responsible for uterine muscle contractibility during labour, and has been used for labour induction and augmentation for decades [13–15]. While slow or no progress in labour, which are associated with increased maternal and fetal morbidity [16], can be remedied with oxytocin augmentation [15, 17], there is no conclusive evidence that augmentation of labour with oxytocin decreases caesarean section rates [16, 18].

The number of oxytocin receptors in the uterus is dynamic and increases as pregnancy approaches term. This leads to a higher uterine sensitivity to oxytocin and causes uterine contractions [19]. In contrast, a reduction in the number of oxytocin receptors has been reported in women receiving oxytocin for induction or augmentation of labour [19]. Oxytocin shows particular pharmacodynamics and kinetics [15]; for example, its effect lasts for approximately 10 minutes, but its half-life decreases when plasma concentrations are increased with the use of oxytocin infusions [20]. According to Clark et al. [21] oxytocin has three distinctive characteristics. First, the desired plasma level and effect is usually reached after 40 min [22]. Second, the safe and optimal dosage is challenging to determine because oxytocin has a variable degree of efficiency [1, 2, 21, 23]. Third, artificially inducing and/or increasing the rate and strength of uterine contractions has an unpredictable effect on fetal oxygen saturation [24].

An intravenous high-dose bolus of oxytocin is recommended immediately after the birth of the anterior shoulder to prevent postpartum haemorrhage [25]. After birth, oxytocin plays an important role in maternal well-being, bonding and lactation [24, 26, 27]. Reduced maternal oxytocin levels and increased prolactin levels during breastfeeding have been reported after in-labour oxytocin infusion (exogenous oxytocin) [24]. In one study, women who were breastfeeding their babies and who received exogenous oxytocin during labour showed reduced levels of anxiety and aggression, traits associated with endogenous oxytocin [27]. In addition, depressed reflexes associated with breastfeeding in newborns and oxytocin infusion during labour have been reported [28, 29]. Consequently, the administration of oxytocin during labour appears to have long-lasting effects on mother and child [30].

Overall, oxytocin has particular pharmacodynamic and kinetic characteristics with versatile effects on the human body [15]. Exogenous oxytocin administration can cause various side-effects, such as hyperactive uterine activity, even uterine rupture, and fetal hypoxia [15, 31, 32]. Studies reporting on women who have intrapartum oxytocin augmentation are very rare [33]. In 2007, the Institute for Safe Medication Practices (ISMP) added oxytocin to the list of high-alert medications, and urged caution with its administration during labour [21, 34]. Consequently, having a clinical guideline on the use of oxytocin for inducing and/or augmenting labour is the first step towards safer administration [18, 31, 35–37], and has the potential to eliminate inter-institutional variations [3] and minimise the risk of administration errors.

There are a number of international and national guidelines and recommendations on oxytocin administration for labour induction and augmentation (summarised in Table 1). These include: the Irish guideline, developed by the Health Service Executive (HSE) on *Oxytocin to accelerate or induce labour* [37]; the National Institute for Health and Care Excellence (NICE) guideline on *Intrapartum care for healthy women and babies* [35]; the Nordic Federation of Societies of Obstetrics and Gynaecology (NFOG) guideline on *Augmentation of labour* [36]; the American Congress of Obstetricians and Gynaecologists (ACOG) practice bulletin number 107 on *Induction of labour* [31], and the French recommendations on *Oxytocin administration during spontaneous labour*, developed by the CNGOF (French National College of Gynecologists Obstetricians) and the CNSF (National College of Midwives) [38]. These guidelines vary regarding the starting dose of international units (U) used; fluid type and volume; minimum, escalation and maximum dosages, and escalation time intervals. Most of the guidelines recommend performing an amniotomy prior to commencing an oxytocin infusion while all guidelines mention uterine hyperactivity as a contraindication for continued use. Further indications and contraindications are given together with defined criteria to start or stop oxytocin infusion during labour. Case-specific recommendations in relation to parity, uterine scars, term, preterm and twin pregnancies are usually provided.

In Germany, there are no guidelines or national data on intrapartum oxytocin augmentation. Due to the limited information, and in order to be able to contribute to a national guideline, this study aimed to describe the most common clinical practice patterns on oxytocin augmentation during labour.

## Methods

### Setting

All 34 university compounds in Germany were surveyed. Five university hospitals have campuses with two separate maternity units, and these were treated as independent units resulting in a total of 39 maternity units. The clinical director of the university gynaecological hospitals, senior physicians and head midwives were contacted by e-mail. All participating units were obstetrician-led units [39].

### The tool

This survey is part of a larger international, unfunded survey, which was initiated during the EU COST Action IS1405 on organizational perspectives (http://www.cost.eu/COST_Actions/isch/IS1405; https://eubirthresearch.eu/). The survey was translated from a 20-item survey that was based on the tool used to capture institutional practices on oxytocin acceleration or induction of labour in Ireland [37, 40]. The questionnaire was originally designed according to the NHSLA/CNST standard 2 criterion 5 of the Maternity Clinical Risk Management Standards. Therefore, it included the following five categories: oxytocin dosage, criteria for oxytocin administration and indications, contraindications, case-specific conditions and the process of developing SOPs. Additional file 1: Table S1 shows the questionnaire and the NHSLA use of oxytocin can be found online (http://workplacehealthandwellbeing.co.uk/publication/syntocinon-infusion-regime-io25v2/). The survey was not piloted but maternity care practitioners in eleven countries, ten European and South Africa, reviewed the content for accuracy and completeness.

### Ethical approval

The study and research protocol were approved by the Ethical Review Board of Hannover Medical School (no. 3317–2016).

### Data collection

The study information, survey and consent forms were sent electronically in August 2016. Participating units could choose to complete the survey or forward their SOP to the research team. Written consent was obtained from all participants. When SOPs alone were received, data were entered into the data collection form by SH. Hospitals’ names were removed.

### Data preparation

Some responses contained more than one answer regarding oxytocin dosages used and titration rates. In those cases, all responses were reviewed. For ease of interpretation and comparison, we converted dosage information to mU/hour. After conversion, the median starting dosage of 120 mU/h correlated to 2 mU/min. In turn, this equated to 10 ml/hour or 0.16 ml/minute when the solution was prepared with 6 U oxytocin in 500 ml of fluid (oxytocin concentration of 12 mU/ml).

### Statistical analysis

Data were analysed descriptively using GraphPad Prism 7. Frequencies were used to summarise categorical data: international oxytocin units, volume, infusion fluid and final oxytocin concentration. Continuous data were summarised using the mean distribution: starting, maximum and escalation dosage.

## Results

A total of 35 out of 39 (90%) maternity units returned the survey and/or SOP.

Twenty-five units (71%) completed the survey alone, six (17%) completed the survey and sent their SOPs and 4 units (11%) sent the SOP alone.

### Development of standard operational procedures

Almost half of the SOPs (44%, *n* = 16) were developed by (senior) consultants (34%, *n* = 12) or the assistant medical director and/or the head of the department of obstetrics (11%, *n* = 4). Three SOPs (9%) were developed by (head) midwives, with input from the assistant medical director or the head of the department. Another three procedures (9%) were developed jointly by (senior) physicians and (head) midwives and 2 units (6%) stated that the entire obstetric and midwifery team developed the SOP.

Midwives participated in the development of SOPs in less than half of the units (43%, *n* = 15), while 9 units (26%) reported no midwifery involvement. No SOP was available in 7 units (20%) and information was missing from 4 units (11%).

### Conditions to start and monitor oxytocin administration for labour augmentation

The main indication given for augmenting labour with oxytocin was delayed progress during labour (31%, *n* = 11). Criteria included prolonged first stage of labour (23%, *n* = 8) or prolonged second stage of labour, delayed progress with or without previous amniotomy (11%, *n* = 4 for each criterion). In another 11% of the units (*n* = 4) the administration of oxytocin was specifically prescribed by a physician, while the criteria for starting oxytocin administration were up to the individual physician (6%, *n* = 2) and women’s written consent was required in 6% of the units (*n* = 2).

After oxytocin infusions were started, continuous cardiotocography (CTG) was used to monitor labour in 23 units (66%). CTG was continued for 30 min in 12 units (52%), while the remaining units stated no further information on CTG monitoring. Three units also used ultrasonography, took fetal blood samples or performed vaginal examination (3%, *n* = 1 for each criterion) to evaluate the obstetric situation. Four units (11%) reported no defined indications or criteria, and data on starting criteria were missing for two units (6%).

Although the survey asked specifically about indications for oxytocin administration to augment labour, additional details on induction of labour was reported by five units (14%), delivery of the placenta by one unit (3%) and accelerating uterine contractions after the birth of the first twin by three units (9%).

### Preparation of oxytocic infusion

**Fig. 1:**
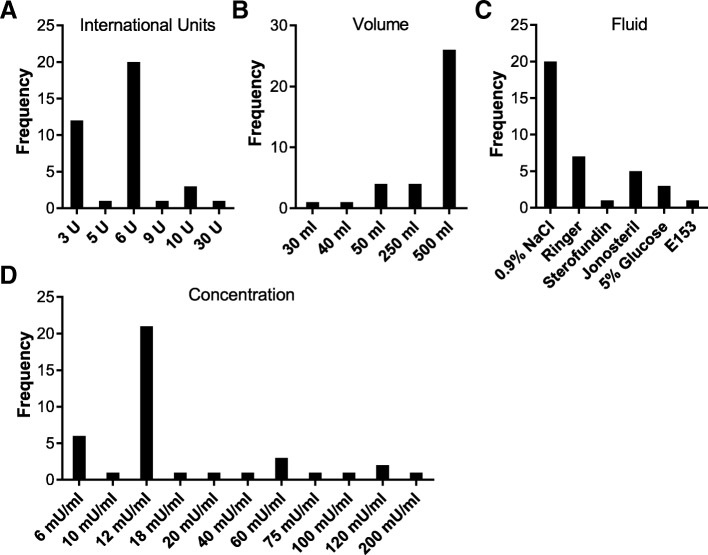
Descriptive analysis of oxytocin preparation and concentration in German university hospitals. The frequencies of used international oxytocin units (**a**), the volume in ml (**b**) and the fluid it was prepared in (**c**) as well as the final oxytocin concentrations in mU/ml (**d**) are depicted. (NaCl: Sodium chloride; E 153: electrolyte infusion solution 153)

The preparation of oxytocin infusions varied in relation to the number of international units used, and the type and quantity of the infusion fluid (Fig. 1). More than 50% of the units (*n* = 20) prepared oxytocin infusions using 6 U while another twelve (32%) used 3 U (Fig. 1a). Three of the SOPs gave two alternative oxytocin dosages (3 U or 6 U), giving a total of 38 responses (total *n* = 35 + 3). The majority of maternity units use a volume of 500mls (72%, *n* = 26) (Fig. 1b). One SOP gave a two-volume option, 250 ml or 500 ml (total *n* = 35 + 1). Normal saline was the most commonly used infusion fluid (*n* = 20, 54%) (Fig. 1c) and two units specified two fluid options, resulting in a total of 37 responses (total *n* = 35 + 2).

When converted to U/ml, the oxytocin concentration in 55% (*n* = 21) of units was set to12mU/ml (Fig. 1d). Overall, the concentration ranged from 6 mU/ml to 200 mU/ml. Two alternative oxytocin dosage regimens in three protocols resulted in additional three data points (total *n* = 35 + 3).

### Oxytocin dosage

**Fig. 2:**
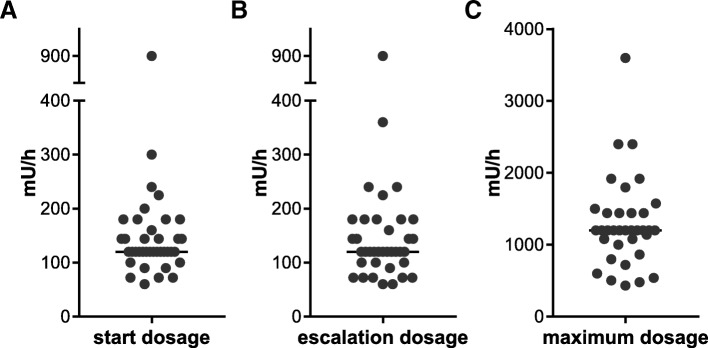
Range of oxytocin dosage for labour augmentation in German university hospitals. This Figure contains the frequencies of starting (**a**), maximum (**b**) and escalation dosage (**c**) in mU/h

Starting, escalation and maximum dosages, as well as escalation intervals, were compared (Fig. 2). The median concentration of the starting dosage and the escalation dosage was 120 mU/h, stated by 37% (*n* = 14/38) and 29% (*n* = 11/38) of units, respectively (Fig. 2a, b). In contrast, the maximum dosage showed greater variation with a median concentration of 1200 mU/h (31%, *n* = 11/35) (Fig. 2c). Escalation intervals varied between 20 (34%, *n* = 12), 15 (17%, *n* = 6) and 30 min (11%, *n* = 4). Only a few maternity units stated a time range, the most common range being 15–20 min (9%, *n* = 3).

### Titration of oxytocin dosage

Oxytocin dosage was titrated with uterine contractions in 29 units (83%). One unit (3%) stated it was not titrated and data were missing from five units (14%). Uterine contractions were monitored by continuous CTG in 26 units (74%) while two units (6%) used intermittent tocometry. In four units, uterine contractions were monitored every 10 min (*n* = 3) or every 30 min (*n* = 1) but the method(s) used were not specified. One unit stated the frequency of monitoring contractions varied (3%). Data were missing for two units (6%).

### Contraindications and criteria for stopping intrapartum oxytocin infusions

The most commonly stated contraindication was a pathological cardiotocograph (CTG), stated by 10 units (29%). Overall, oxytocin administration was contraindicated when vaginal birth and/or uterine contractions were contraindicated (20%, *n* = 7). These included the following contraindications: cephalopelvic disproportion and mechanical obstruction, placenta or vasa praevia, active herpes genitalis, abruption of the placenta, abnormal lie or position of fetus (e.g. transverse), maternal heart defects/problems, prolapse of the umbilical cord as well as a suspected fetal asphyxia.

Further contraindications included uterine hyperactivity (17%, *n* = 6) and having had a previous caesarean section or any further uterine surgery (14%, *n* = 5), risk for uterine rupture and fetal heart rate abnormalities or problems (both 11%, *n* = 4).

Several units gave multiple responses. Four maternity units gave no contraindications for oxytocin (11%), and data were missing from another four university hospitals (11%).

The criteria for stopping oxytocin infusion were grouped according to reasons: fetal (83%, *n* = 29), placental (17%, *n* = 6), uterine (69%, *n* = 24), maternal (17%, *n* = 6) and others (26%, *n* = 9). Overall, a pathological CTG and hyperstimulation of the uterus were the most frequently given criteria, stated by 21 units (60%), and another 10 units (29%) gave shoulder dystocia as a criterion. Data were missing from 3 units (9%).

In summary, a total of 30 different contraindications for oxytocin augmentation were reported and 35 criteria for discontinuing oxytocin infusion. Multiple answers were given in most cases.

### Alterations in oxytocin administration in case-specific circumstances

Oxytocin administration regimens can differ depending on case-specific conditions such as parity and previous uterine surgery. In this study, no case-specific recommendations were reported for primiparous and multiparous women in any unit, or for women in preterm labour in 66% of units (*n* = 23). Women with a twin pregnancy were advised to receive an oxytocin infusion to augment labour according to standard protocols in 49% of units (*n* = 17).

Protocols varied for women with uterine scars: 37% (*n* = 13) of the units used lower oxytocin dosages, slower escalation rates or reduced maximum dosages. Among others, additional recommendations were monitoring with CTG (14%, *n* = 5), consultation with an obstetrician (6%, *n* = 2), ultrasonography of the uterine scar or delaying the oxytocin infusion until later in labour (both 3%, *n* = 1).

Recommendations for women with twin pregnancies included restrictive administration of oxytocin (12%, *n* = 4) before, and increase in oxytocin, administration after the birth of the first twin (9%, *n* = 3), and oxytocin administration in combination with CTG monitoring (6%, *n* = 2).

Individually adjusted recommendations were stated for approximately 10% of case-specific alterations.

## Discussion

This study demonstrated considerable variation in the intrapartum oxytocin infusion regimens used in German university hospitals. Regimens differed widely in several aspects, including indications for administration and preparation of oxytocin solutions. Overall, most university hospitals prepared oxytocin solutions with 6 U (53%, *n* = 20), in 500 ml of intravenous fluids (72%, *n* = 20), which resulted in an oxytocin concentration of 12 mU/ml. The most commonly used infusion fluid was 0,9% NaCl (54%, *n* = 20). These results fall into the ranges reported in the HSE, NICE, NFOG and ACOG guidelines: 5-30 U in 500-1000 ml NaCl or Ringer lactate, but they differ in the exact values and infusion fluids (Table 1) [31, 35–37]. We identified varying indications, contraindications and case-specific guidelines for intrapartum oxytocin administration in most of the maternity units. Moreover, our study depicts the magnitude of inter-institutional variations for augmenting labour using oxytocin [3]. Although there is no recommendation on the superiority of one regimen over others [1, 2], such variations pose challenges for clinicians, the mother and the unborn as well as the reproducibility of favourable outcomes.

**Table 1:** International guidelines for the administration of oxytocin

	HSE (Ireland)	NICE (Great Britain)	NFOG (Denmark, Sweden, Finland, Iceland, Norway)	ACOG (USA)	Empiric study on German university units (*n* = 35)
International Units (U)	10	/	5	30	6
Oxytocin solution	1 l NaCl	/	500 ml NaCl	500 ml Ringer lactate	500 ml NaCl
Oxytocin concentration (mU/ml)	10	/	10	60	12
Start dosage (mU/h)	60–300	/	360	120	120
Maximum dosage (mU/h)	1800	4–5 contractions/ 10 min	2400	9000	1200
Escalation dosage (mU/h)	60–300	/	180	120	120
Time interval (min)	15–30	30	15	30	20
Monitoring	CTG	CTG	CTG	CTG	CTG
Criteria for oxytocin administration	20 min CTG, stable fetal status, preceding amniotomy	CTG, preceding amniotomy	CTG, preceding amniotomy	Stable fetal and maternal status, continuous monitoring	CTG, monitoring of obstetric situation
Indication	Slow labour, reduced contraction frequency	Slow labour, reduced contraction frequency	in-effective contractions	Induction and augmentation of labour	Labour augmentation
Contra-indications	Fetal distress, hyperactive uterus, uterus scar, fetal malposition	Hyperactive uterus	Hyperactive uterus, shoulder dystocia	Hyperactive uterus, water intoxication, fetal distress, no monitoring possible	Pathological CTG, hyperactive uterus, shoulder dystocia
Case-specific variations	Multipara, uterus scar, pre-term labour, twin pregnancy, maternal heart insufficiencies	Multipara, regional analgesia	Sensitivity is individual for every women and the administration should be adapted accordingly, uterus scar	After amniotomy reduction of oxytocin dosage, guideline for: singletons, vertex, in term, without uterus scars	Uterus scar, twin pregnancy (multipara, pre-term)

To identify similarities and differences with other countries, we compared our findings with the international guidelines in relation to indications for starting augmentation; monitoring uterine activity during labour augmentation; infusion solutions and oxytocin dosage; contraindications for use, criteria for stopping the infusion and criteria for alterating the regimen.

### Conditions to start and monitor oxytocin administration for labour augmentation

The main focus of the study was the intrapartum administration of oxytocin for labour augmentation. The 35 maternity units that participated in our study stated delayed progress of labour was the main indication for augmentation with oxytocin, but there was no clear definition of delayed progress in labour. Differing criteria can be also found in other guidelines. For example, NFOG advises oxytocin acceleration when cervical dilatation is less than 1 cm/hour after 2 hours in the active phase in nulliparous women, and after 4 hours in multiparous women [36]. According to NICE (2017), delayed labour progress is assessed by 1) cervical dilatation of less than 2 cm in 4 h in a first labour, 2) cervical dilatation of less than 2 cm in 4 h or a reduction in labour progress for second or subsequent labours, 3) delay in descent and rotation of the fetal head, 4) and changes in the strength, duration and frequency of uterine contractions [35].

Oxytocin augmentation is one component of the active management of labour package which was introduced in Dublin in the 1960s [41]. Labour progress is different in nulliparous and multiparous women, differences which have been acknowledged already for decades [42]. The normal progress of labour was investigated by Friedman [43] and re-evaluated more recently by Zhang [44, 45]. Recent studies on the use of the partogram to monitor fetal and maternal key data during labour showed that they can minimise the administration of oxytocin without impacting negatively on progress of labour in terms of frequency of operative births and safety criteria such as the Apgar score [46]. This is especially interesting as the administration of oxytocin alone did not reduce the need for operative births [46, 47], in contrast to early amniotomy and oxytocin augmentation together [48]. It is still not clear if delayed oxytocin administration would contribute to an increase in spontaneous birth [49]. In recent years, research on oxytocin focussed on negative effects on children’s development. For example, synthetic oxytocin was suspected to effect hyperactivity/inattention problems in children, even though the data did not support the hypothesis [50].

### Preparation of oxytocin infusion, dosage and titration

The most frequently used starting dosage was 120 mU/h with a median maximum dosage of 1200 mU/h. The most frequently used escalation dosage was 120 mU/h, and the most frequently used escalation interval was 20 minutes. Both starting and escalation dosages correlate with the ACOG guideline but the median maximum dosage in German university hospitals is lower compared to all four guidelines (Table 1) [31]. A Cochrane review found a reduced labour duration and increase in spontaneous births when a higher starting and escalation dosage was used [2]. This was defined as 4 mU/min or more, which is achieved with 6 U oxytocin in 500 ml NaCl and a starting and escalation dosage of 20 ml/h. This regimen was only recommended by NFOG and was included in the range of dosage in the HSE guidelines [36, 37]. No increased side effects of high oxytocin starting dosages were found (instrumental vaginal birth, epidural analgesia, hyperstimulation of the uterus, postpartum haemorrhage, chorioamnionitis, women’s perceptions of experiences, Apgar scores, umbilical cord pH, admission to special care baby unit, neonatal mortality) [2]. Nevertheless, the review authors concluded that they could not give a general recommendation for a high-dose regimen because of insufficient evidence [2].

The escalation interval of 20 min lies in the recommended range of 15–30 min. Nevertheless, oxytocin has been shown to reach the desired plasma level after approximately 40 min [20] and dosage should only be increased after 30 min or later according to NICE and ACOG [31, 35]. Hence, variation in the escalation time interval may increase unintended side effects.

The ACOG guideline recommends fetal heart rate observation using continuous electronic CTG for 20–30 min prior to starting oxytocin administration, and the HSE guideline states CTG should be performed for at least 20 min [31, 37] which is similar to our findings (Table 1). A total of 23 maternity units (66%) monitored the fetal heart rate using CTG; 52% (*n* = 12) for a duration of 30 min and 9% (*n* = 2) for 20 min. Oxytocin dosage was titrated with uterine contractions in 83% (*n* = 29) of the units, and uterine activity was monitored with continuous CTG in 74% (*n* = 26). Infusion titration with uterine contractions minimise the risk and consequences of uterine hyperstimulation. The NICE guideline recommends achieving 4–5 contractions in 10 min [35] and NFOG recommends a rate of 3–5 in 10 min [36].

Three out of four guidelines recommend performing an amniotomy before starting an oxytocin infusion. In our survey, only 4 units (11%) gave previous amniotomy as a criterion to start oxytocin (Table 1). Previous studies comparing the use of amniotomy to no treatment showed a reduced labour duration of two hours, but no reduction in the number of caesarean sections performed [16]. In a review of 11 trials, performing amniotomy prior to oxytocin administration was associated with a modest reduction in the number of caesarean births [51], and labour duration was reduced by an average of 1.28 h (95% CI − 1.97 to − 0.59; eight trials; 4816 women) [51]. Hence, the impact of a combined use of amniotomy and oxytocin on labour progression and caesarean section rates needs to be investigated further.

### Contraindications and criteria for intrapartum oxytocin cessation

The recommendations for oxytocin administration in existing guidelines vary depending on the women’s specific conditions. Hyperstimulation is defined as more than 7 uterine contractions in 15 minutes in nulliparous women and more than 5 contractions in 15 minutes in multiparous women, according to HSE guideline [37]. The ACOG guidelines define more than 5 contractions in 10 minutes, averaged over a 30-min interval [31]. NFOG defines hyperstimulation as more than 5 contractions in 10 minutes or a duration of the contraction with more than 2 minutes [36]. Not all units used CTG monitoring, but four international guidelines recommended its use to detect fetal heart rate changes, a contraindication to starting and a criterion for stopping oxytocin infusion in most of the units (83%; Table 1).

Further contraindications included general contraindications for vaginal birth. These criteria can also be found in international guidelines, such as ACOG (placenta/vasa praevia, transverse fetal lie, umbilical cord prolapse, previous classical caesarean delivery, active genital herpes infection, previous myomectomy entering the endometrial cavity) [31].

### Alterations in oxytocin administration in case-specific circumstances

Several units stated that oxytocin should be used with caution when there was a history of previous uterine survey. This is supported by studies which showed an increased dose-dependent risk of uterine rupture with oxytocin [52]. A maximum dosage of 20 mU/min was recommended, achieved at a rate of 100 ml/h when 6 U oxytocin is added to 500 ml NaCl [52]. Specific guidelines for management of vaginal birth after caesarean section (VBAC) did not include a maximum dosage [53, 54]. However, two guidelines advised clinical assessment by a senior obstetrician before augmentation during the first stage of labour [35, 37]. In addition, the HSE guideline does not recommend oxytocin administration use in the second stage of labour in women with uterine scars [37].

Recommendations for women with twin pregnancies included a restrictive administration of oxytocin before (9%, *n* = 3), and an increase in oxytocin dosage after (11%, *n* = 4), the birth of the first twin. These results are in line with the hypothesised increase of contraction frequency and overall response to oxytocin with increased birth weight and degree of tissue stretching during a twin pregnancy [23]. A restrictive administration at the start of the infusion reduces the risk of oxytocin overdose and associated side-effects while the dosage can be increased after the birth of the first twin when the uterine size and consequently the level of stretch decreases and an increase in uterine contractions is desirable. Only the HSE guidelines mentioned twin pregnancies and recommended CTG monitoring for both twins and starting oxytocin when prescribed by a senior obstetrician [37].

Furthermore, both the HSE and NICE guidelines advise restrictive use of oxytocin in multiparous women, and the NICE guideline states that an obstetrician should perform a full assessment and a vaginal examination before the decision to use oxytocin is made [35].

Four units reported restricted use of oxytocin administration in preterm labour. This is supported by the HSE guideline, which in general advise caution with oxytocin [37].

## Limitations

There were a number of limitations to our study. It was designed to describe current practices in German university hospitals and compare these with international guidelines. We did not collect additional hospital-level data; therefore, we were unable to explore multiple associations between variables.

Our study is based on a national cohort and data acquired in Germany may not be applicable to other countries. The statistical analyses performed are limited due to the small number of hospitals included. In addition, there may be a selection bias as only university hospitals were asked to participate, and the population of childbearing women in these hospitals may differ from those in other hospitals. Care and practice may also differ in other German non-university maternity hospitals. However, to contextualise the findings, we compared practices with other international guidelines.

One further limitation exists because of missing data from some units, especially on contraindications for oxytocin administration. While it can be suggested that contraindications for spontaneous labour are also contraindications for oxytocin augmentation, units did not report on these in the survey or include them in their SOPs.

The French recommendations for the use of oxytocin during spontaneous labour were published after this study had been finalised [38]. These recommendations were developed jointly by the CNGOF (French National College of Gynecologists Obstetricians) and the CNSF (National College of Midwives) and used in 2018 by the HAS (High Authority of Health) in guidelines on normal birth. In future studies this guideline should be included in the overall evaluation process [38].

## Conclusion

Our study showed a wide variation in oxytocin administration and also in the completeness of the SOPs in the hospitals surveyed. Labour wards, including midwife-led units, should have SOPs, which state the infusion solution, starting and escalation dosage of oxytocin, and indications and contraindications. Protocols should identify procedures to be followed in specific circumstances (VBAC, twins, etc.) to improve safety for women and babies. The development of a national guideline would help midwives and obstetricians to adopt them for their hospital-specific SOP and daily practice, and eliminate inter-institutional variations. CTG monitoring before (for 20-30 min) and during oxytocin infusion should be considered standard, while performing an amniotomy before starting an oxytocin infusion needs further investigation. Overall, a national guideline would help clinicians clinicians develop clear local guidelines on the criteria for oxytocin augmentation of labour, and thereby help minimise medication and administration errors.

## Additional file

Additional file 1: **Table S1.** The questionnaire was used in the national survey: Inter-institutional variations in oxytocin augmentation during labour in German university hospitals. It was originally designed according to the NHSLA/CNST standard 2 criterion 5 of the Maternity Clinical Risk Management Standards. Therefore, it includes the following five categories: oxytocin dosage, criteria for oxytocin administration and indications, contraindications, case-specific conditions and the process of developing SOPs. (XLSX 11 kb)

## Data Availability

All data generated or analysed during this study are included in this published article. The anonymised, original datasets used during the current study are available from the corresponding author on reasonable request.

## Abbreviations

ACOG: American Congress of Obstetricians and Gynaecologists
AVP: Arginine vasopressin
CTG: Cardiotocography
HSE: Health Service Executive
ISMP: Institute for Safe Medication Practices
NFOG: Nordic Federation for Societies of Obstetrics and Gynaecology
NICE: National Institute for Health and Care Excellence
SOP: Standard operating procedures
U: International units
VBAC: Vaginal birth after caesarean section
